# Gleanings in Science and Medicine

**Published:** 1893-07-29

**Authors:** 


### GLEANINGS IN SCIENCE AND MEDICINE.

At periods of long and unwonted drought like the present, the public has fully recognised the necessity of the liberal use of disinfectants. To meet this great demand the disinfectant produced by Jeyes' Sanitary Compound Company has proved itself a valuable commodity. Reliable testimony to this effect has been produced by the Secretary to the Royal Tournament at the Agricultural Hall, as well as the Imperial Institute and the Bisley camp authorities. The disinfectant which has thus been extensively used and tested for seven years, has received medical testimony that it is a real and active germicide, is volatile, and non-poisonous, and, therefore, free from any caustic action on the skin, and is readily soluble in water.

The study of medicine in America continues to receive considerable encouragement. The Chicago College of Physicians and Surgeons has offered no less than ten scholarships each of £100 a year. Although in principle this encouragement is satisfactory to those who are desirous of entering the profession, yet it might fairly be forgiven those already installed therein, were they to ask whether a risk may not thereby eventually be run of bringing about a plethora of doctors! This, of course, would only be speaking from a one-sided and selfish standpoint, but it is a fact that many large towns now do point this out, that there is already an overstocking of the profession. Naples, for instance, possesses a medical practitioner for every 513 of its inhabitants, and this state of things necessarily lessens the chances of any one member earning any substantial means of subsistence.

Following on the discovery that monkeys have speech, we may further be led to anticipate as a possibility the day when speech is also discoverable in the floral as in the animal kingdom. Plants, from the days of Darwin have been credited with the organ of sight (anyhow parasitic and climbing ones), and a weekly contemporary in discussing this subject, speaks with considerable certainty upon it, and we are assured that the fact is based on testimony sufficient to convince the most sceptical that it is more than the mere speculation of a frenzied brain. Before the march of human intellect many cherished sentiments must give way. Nature's hold upon us, so far, has been in her character as an insenate surrounding; to clothe her with attributes formerly supposed to be confined to humanity is to supplement the positive for the negative, and is, alas! to change her from the dispassionate witness of our actions, into the conscious spectator of them.

The Commissioners on Lunacy have lately given us more than the bare outline of facts, categorically arranged, as to how many inmates each asylum possesses, and their age and sex. Besides the food for their bodies the Commissioners have wisely pointed out that the inmates of asylums require food for their brains. All lunatics are not idiots; and yet in the case of one institution visited by these inspectors, only a few odd volumes of books were discoverable in the female wards, whilst in the male ward the one printed *pièce de résistance* for readers was "The Careful Housemaid." This same institution, whilst it registers its 90 patients, could boast of only one copy of a daily newspaper. It is lamentable to think of such a state of things in establishments where social intercourse and rational conversation is necessarily somewhat restricted. It would not cost us much, and it would be a boon to many a public asylum if we would post from time to time odd numbers of illustrated papers or periodicals to while away the long leisure of the inmates.

An excellent method of deodorising iodoform has been furnished by Dr. W. Washburn, of New York. His recommendation is to use ether or chloroform to wash the hands with, both of which act as solvents. The odour of iodoform is of a particularly trenchant character, and clings to the skin in a disagreeable manner. Chloroform applied to hands or clothing has a like beneficial effect in removing all traces of this.

Were the funeral customs of the world to be collected we should find little that could be called an improvement or reform amongst them from early ages to this. Each nation has its own accepted formula characteristic of its people. Our own country presents a curious inconsistency between the spirit and the practice of Christianity as exhibited at the last solemn rites of a departed soul; we sing hymns and psalms, full of resignation and of peace, but we drape the hearse enveloping the remains of the departed soul in deepest black. The life of the Russian peasant has little exemplary about it; it drags its existence away in a degraded slavery to a despotic master, but though his life presents little of the ideal, the death of the peasant in the heart of the Russian country has no equal in its serenity, its peace. To a simple minded, ignorant son of the soil death brings a release from a life of suffering by them to be hailed as a kind deliverer, not only in words, but in the actual spirit.

The example which England has set in its keeping of Sunday is fast being imitated by other countries. In Prussia, for instance, all shops are closed, and business over by twelve a.m., according to the new regulations of the Emperor, who thus enforces a salutary holiday amongst the shop classes. In Belgium a society has arisen which has yet stronger measures in view against the compulsory work of the Government officials on Sunday. The first measure the society is taking is with regard to post office workers. These employés they declare to be overworked, and requiring a greater rest once during the week. An ingenious stamp has been invented on which a tiny flap is affixed, bearing in French and in Dutch the words "This letter is not to be delivered on Sundays." These stamps will be supplied by the General Post Office at the usual letter rate, but the sale would have to be on an extensive scale before it brought about the much desired rest for the postmen on Sundays.

Among the varied caprices of the coming autumnal fashions, the order has gone forth that all the most stylish hats and bonnets are to be decorated with that particular plume known in the millinery world as the aigrette. The dictates of fashion are omniscient, but at her shrine many a sacrifice is entailed, of which her worshippers know little. Not long since, at a protest meeting of certain members of the Anti-Vivisectionist Society, it was noticed that many of the lady members came adorned in all the glory of the aigrette plume. In fairness to them it is only to be assumed on the ladies' behalf that they did not themselves recognise the inconsistency of thus complaining against a body of men for sacrificing dumb animals on the altar of science, when they were fellow torturers themselves on the sacrificial altar of personal vanity. For the way in which the aigrette is obtained entails especial torture to the bird itself; it means a lingering death of pain. Many men are engaged in the trade of collecting, and the ruthless massacre is confined to mother birds, who are on their nests with their young just hatched. The egret is seized, bereft of her plume and her wings, and then flung back on her nest to die.

